# Direct Synthesis of Graphene Dendrites on SiO_2_/Si Substrates by Chemical Vapor Deposition

**DOI:** 10.1186/s11671-020-3245-y

**Published:** 2020-01-17

**Authors:** Yingxian Li, Zhenhua Li, Qingbo Li, Meng Tian, Chunhui Li, Li Sun, Jihua Wang, Xian Zhao, Shicai Xu, Fapeng Yu

**Affiliations:** 1 Shandong Key Laboratory of Biophysics, Institute of Biophysics, College of Physics and Information, Dezhou University, Dezhou, 253023 People’s Republic of China; 2 Institute of Crystal Materials, Advanced Research Center for Optics, Shandong University, Jinan, 250100 People’s Republic of China

**Keywords:** Graphene dendrites, SiO_2_/Si substrates, CVD, Nanoelectronic applications

## Abstract

The long-standing interest in graphene has recently brought graphene-derived materials including graphene hydrogel, graphene fiber and graphene paper into sharp focus. These graphene-derived materials show outstanding properties in mechanics and physics. In this paper, for the first time, we demonstrate the novel synthesis of graphene dendrites on SiO_2_/Si substrates by chemical vapor deposition. The tree-like graphene dendrites with well-controlled morphology can be directly grown on both the Si and the SiO_2_ surfaces of the substrates by using methane and hydrogen as precursors. The graphene dendrites on SiO_2_/Si substrates can be directly used in the fabrication of the electronic device. The conductivity and the Hall mobility of graphene dendrites are ~ 286 Scm^−1^ and ~ 574 cm^2^(Vs)^−1^, respectively. Young’s modulus of graphene dendrites is up to 2.26 GPa. The developed method avoids the need for a metal substrate and is scalable and compatible with the existing semiconductor technology, making graphene dendrites be very promising in nanoelectronic applications.

## Introduction

Graphene is a kind of two-dimensional (2D) crystal material with sp^2^ carbon atoms arranged in a honeycomb lattice. Because of the excellent physical and chemical properties, graphene has attracted tremendous attention since it was found by mechanical cleavage of highly ordered pyrolytic graphite (HOPG) in 2004 [1]. To date, graphene has been demonstrated to be a very promising material in supercapacitors, solar cells, sensors, and so on [2–10]. At the same time, the graphene-derived materials such as one-dimensional graphene fiber, two-dimensional graphene paper, and three-dimensional graphene hydrogel have also been studied extensively. These graphene-derived materials show novel mechanical and electrical properties quite different from graphene[11–14].

Graphene dendrite is a new type of graphene-derived material, which has a tree-like crystal structure. Generally, the dendrite structure can be formed in a non-equilibrium state during the crystal growth process. To date, a variety of materials such as metal, alloy and metal oxide have been demonstrated to form the dendrite structures [15–18]. These dendrites usually have unique physical and chemical properties, making them have wide applications in many fields. For example, the large specific surface area of the dendrite can enlarge the number of active adsorption sites, which is expected to achieve higher sensitivity for the chemical sensors and biosensors [19–21].

Though the dendrites are the ubiquitous crystal form in freezing alloys and super-cooled melts, the graphene oxide dendrite and graphene dendrite are not synthesized until 2015 [22, 23]. The graphene oxide dendrite was first synthesized by a chemical reaction in several steps, which was demonstrated to be useful in sensing and separation [22]. In the same year, Liu et al. prepared the graphene dendrite by electrochemical reaction using graphene oxide as the precursor. The prepared graphene dendrite showed a conductivity of 44 Sm^−1^ and was used as the electrode in supercapacitors [23]. However, up to now, the synthesis of graphene dendrites is limited to electrochemical reaction using graphene oxide as the precursor. The conductivity of the synthesized graphene dendrites is still relatively low due to the poor conductance of graphene oxide. Moreover, a metal substrate is indispensable in the electrochemical reaction. As a result, the complicated and skilled post-growth techniques have to be employed to remove metal substrates and transfer the graphene dendrites to dielectric substrates (SiO_2_/Si or quartz) for fabricating electronic devices [24–26].

In this work, we develop a facile strategy to directly fabricate graphene dendrites on SiO_2_/Si substrates by chemical vapor deposition (CVD) using methane and hydrogen as precursors. Without using any catalyst, the tree-like graphene dendrites with high density were directly grown on dielectric substrates. This method does not require the metal substrate, and thus is compatible with the fabrication process of electronic device. The fabricated graphene dendrites show a good conductivity of ~ 286 Scm^−1^, which is about 6.5 times higher than that synthesized by the electrochemical method. The Hall mobility of the graphene dendrites is up to ~ 574 cm^2^(Vs)^−1^ by the Hall effect measurement. Moreover, the graphene dendrites show excellent mechanical properties with Young’s modulus up to 2.26 GPa. The developed technique is compatible with the existing semiconductor technology, thus will be very useful in nanoelectronic applications such as biochemical sensors, nano-electromechanical systems, as well as molecular electronics.

## Methods

### The Growth of Graphene Dendrites

**Fig. 1:**
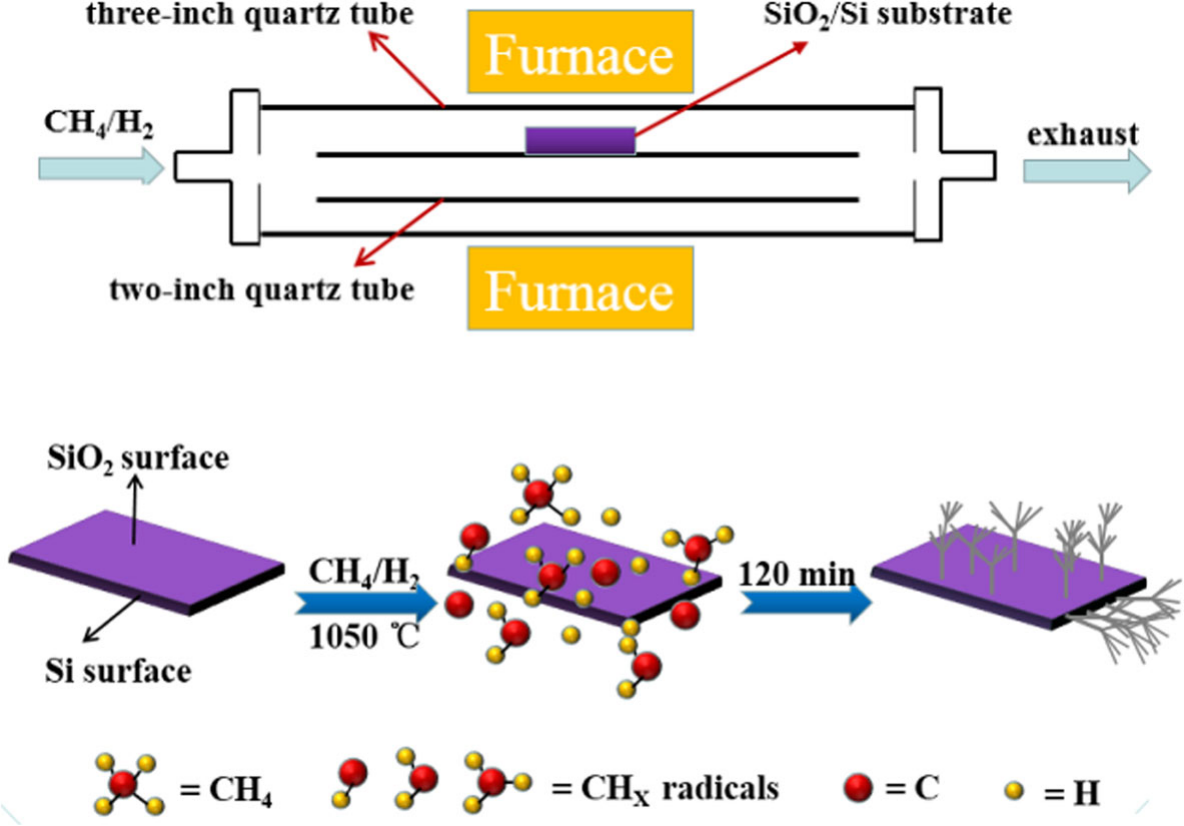
Schematic illustration of CVD process for growing graphene dendrites

N-type Si wafers with a 300 nm thickness SiO_2_ layer were used as the substrates. These substrates were sequentially cleaned by acetone, anhydrous ethanol, and deionized water before they were loaded into the CVD reaction system. The detailed experimental apparatus and experimental process are schematically illustrated in Fig. 1. The cleaned substrates were placed on the top-surface of the outer wall of a 2-in. quartz tube, and then the 2 in. quartz tube was placed inside a 3-in. quartz tube in the CVD chamber. The precursor CH_4_ and H_2_ were introduced into the CVD reaction system, following the vacuum reached as low as 1 × 10^−4^ mbar. The flow rate of CH_4_ and H_2_ was 25 sccm and 15 sccm, respectively. As the temperature was increased to more than 950 °C, CH_4_ began to decompose and deposit on the substrates thus acting as the carbon source for the graphene dendrites. When the growth process was completed, the samples were rapidly cooled down to room temperature at a rate of ~ 100 °C/min by exposing the cube in air. The gas emissions from the CVD reaction system were burned in a pyrolysis furnace and then discharged into the air.

### Characterizations

The morphology of the graphene dendrites on SiO_2_/Si substrates was characterized utilizing the scanning electron microscopy (SEM, ZEISS, SUPRATM-55). The energy dispersive spectroscopy (EDS) was applied for element analysis of graphene dendrites. X-ray photoelectron spectroscopy (XPS) spectra of the samples were measured using a Thermofisher ESCALAB 250 with monochromatized Al Kα X-ray radiation. The as-grown graphene dendrites were evaluated using a confocal Raman spectroscopy (LabRAM HR800) in a backscattering configuration with a 532 nm laser for excitation. The selected area electron diffraction pattern (SAED) and transmission electron microscopy (TEM) images of graphene dendrites were acquired using transmission electron microscopy (TEM, JEOL JEM2100) with an acceleration voltage at 200 kV. The electrical properties (*I*_SD_-*V*_SD_ characteristics) were measured with a semiconductor parameter analyzer (PDA FS360) coupled with a probe station (PEH-4) at room temperature. The mechanical properties of the graphene dendrites were characterized by Atomic force microscopy (AFM, Bruker Multimode 8) in PeakForce Quantitative Nanomechanical Mapping (PFQNM) mode in air.

## Results and Discussion

**Fig. 2:**
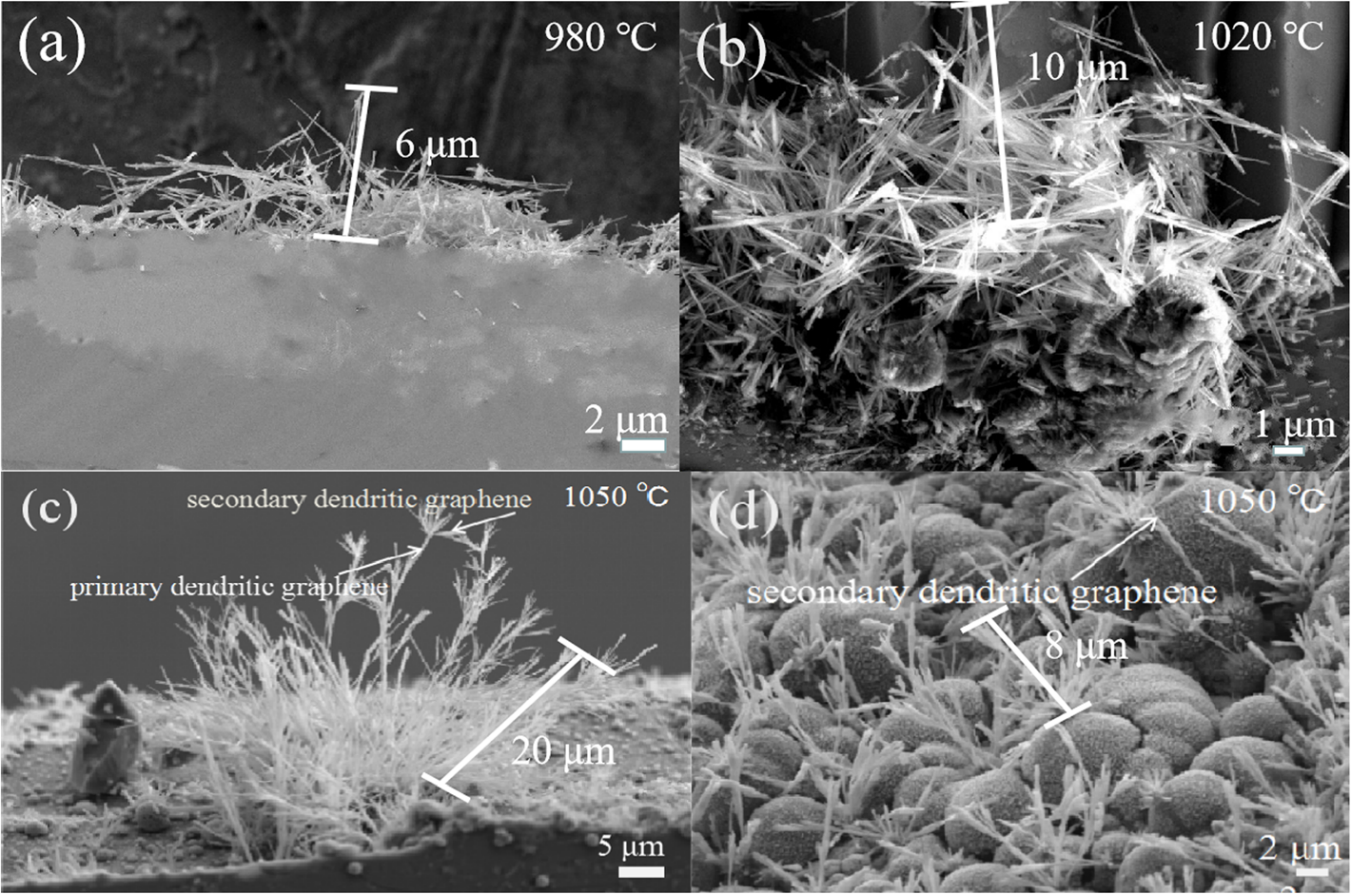
SEM images of graphene dendrites synthesized on Si surface of the SiO_2_/Si substrates at the growth temperature of 980 °C (**a**), 1020 °C (**b**), and 1050 °C (**c**), and on SiO_2_ surface of the SiO_2_/Si substrates at 1050 °C (**d**). The growth time was 120 min

The graphene dendrites were synthesized on the SiO_2_/Si substrates using a low-pressure CVD system. In this CVD system, the growth parameters of the graphene dendrites can be precisely controlled. Figure 2a–c show graphene dendrites grown on the Si surface of the SiO_2_/Si substrates at a different temperature from 980 °C to 1050 °C. The growth temperature can greatly affect the configuration and the length of the graphene dendrites. As can be seen in Fig. 2, the graphene dendrites grown at 980 °C are high density and the typical dendrite length is about 6 μm (Fig. 2a). For the graphene dendrites grown at 1020 °C, the typical length of the graphene dendrites is about 10 μm (Fig. 2b). When the growth temperature further increases to 1050 °C, the total length of graphene dendrites increases to about 20 μm (Fig. 2c). Interestingly, we find that graphene dendrites grown at 1050 °C show a typical tree-like structure with many secondary dendrites grown on the top of the primary dendrite structure. The tree-like graphene dendrites also can be grown on SiO_2_ surface of the SiO_2_/Si substrates at 1050 °C as shown in Fig. 2d. The length of the tree-like graphene dendrites is typically less than 10 μm. As shown in Fig. 2c, d, graphene dendrites grow along a certain direction, which can be attributed to the anisotropic surface energies of SiO_2_/Si substrate [27].

**Fig. 3:**
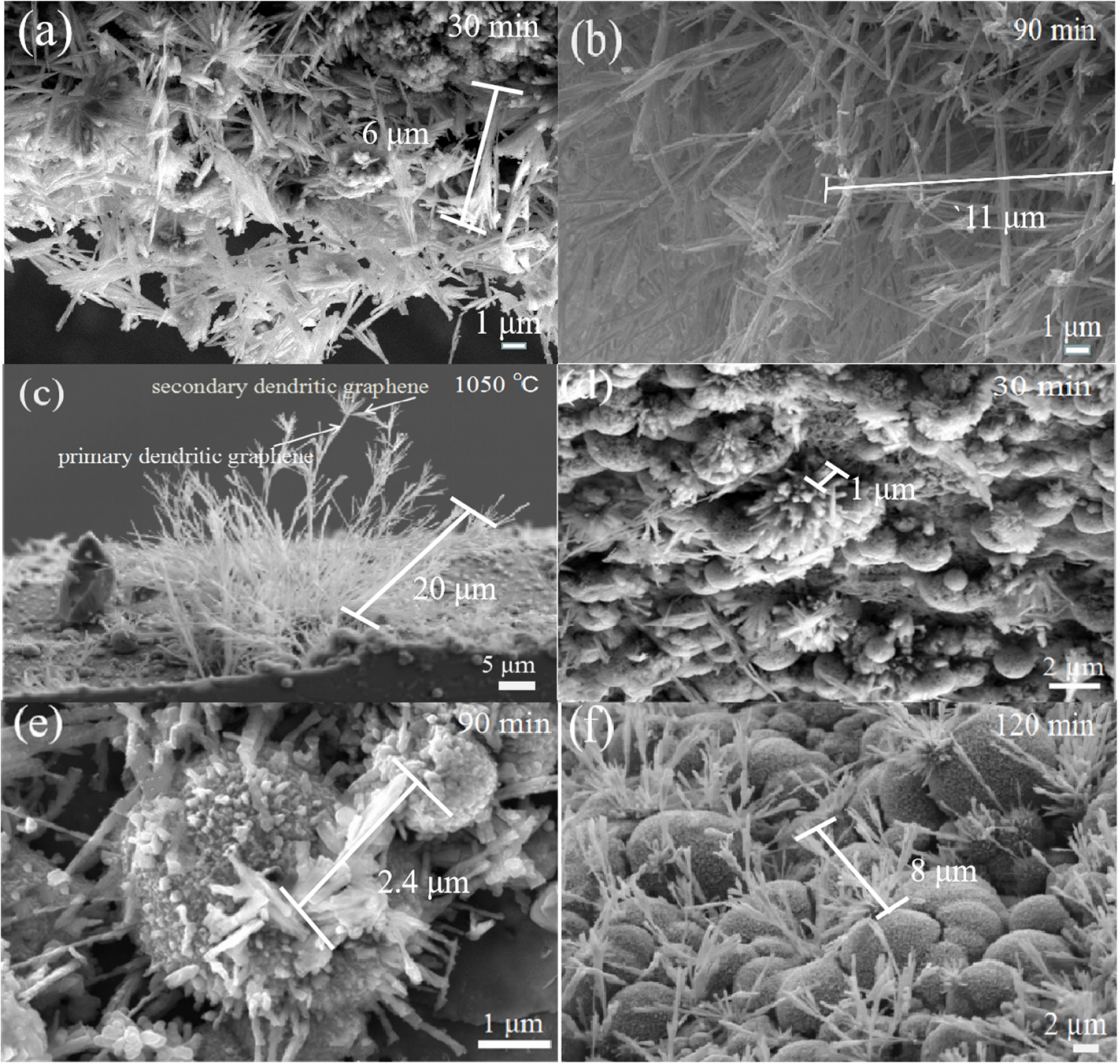
SEM images of tree-like graphene dendrites synthesized on the Si surface (**a–c**) and on the SiO_2_ surface (**d–f**) of the SiO_2_/Si substrates for the different growth time from 30 to 120 min at 1050 °C

The morphologies of the graphene dendrites are also strongly affected by the growth time. Figure 3 shows SEM images of the graphene dendrites grown at different growth time. As the growth time increases from 30 to 120 min, the length of the tree-like graphene dendrites on the Si surface increases from ~ 6 to ~ 20 μm (Fig. 3a–c), and the length of the dendrites on the SiO_2_ surface increases from ~ 1 to ~ 8 μm (Fig. 3d–f). As can be seen from Fig. 3, the length of dendrites grown on the Si surface is larger than that grown on the SiO_2_ surface under the same growth conditions. This phenomenon can be attributed to the fact that the roughness of the Si surface is larger than that of the SiO_2_ surface, as a polishing process was performed on the SiO_2_ surface. Generally, the rough substrate has large surface energy [28, 29], which is beneficial for the growth of the graphene dendrites. Moreover, a hetero-structure is expected to form between graphene dendrites and Si surface, as the work function of graphene (4.5~4.8 eV) is higher than that of Si (~ 4.3 eV), which allows electron charge transfer from Si to graphene [30–32].

**Fig. 4:**
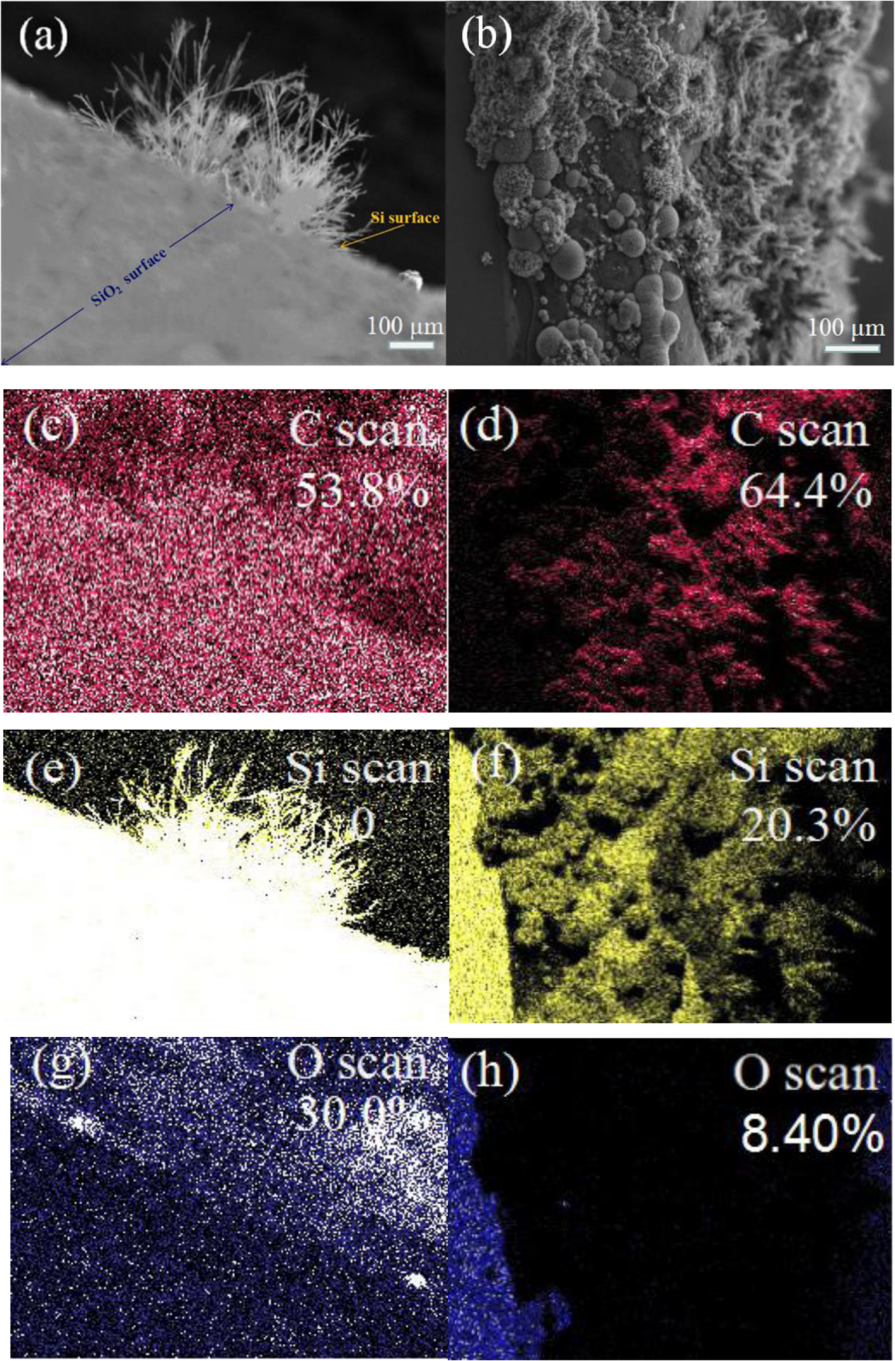
SEM images of graphene dendrites grown on the Si surface (**a**) and SiO_2_ surface (**b**). EDS maps of element content of C (**c**), Si (**e**), and O (**g**) scanned in the same area with (**a**). EDS maps of element content of C (**d**), Si (**f**), and O (**h**) scanned in the same area with (**b**)

The elemental composition of the samples grown on both Si surface and SiO_2_ was investigated by EDS. Figure 4a, b show the SEM image areas performed by EDS on both Si and SiO_2_ surface, respectively. The EDS maps for the elemental C, Si and O of the samples are shown in the Fig. 4c–h. The percentages of the elemental content of the structures are labeled in the right-top EDS scan maps. On both Si and SiO_2_ surface, elemental C dominates with over half that 53.8% on Si surface and 64.4% on SiO_2_. A small amount of Si and O elements are also observed (Fig. 4e–h), which are considered to come from SiO_2_/Si substrates. The EDS result confirms that the elemental composition of the sample is in accordance with that of graphene.

**Fig. 5:**
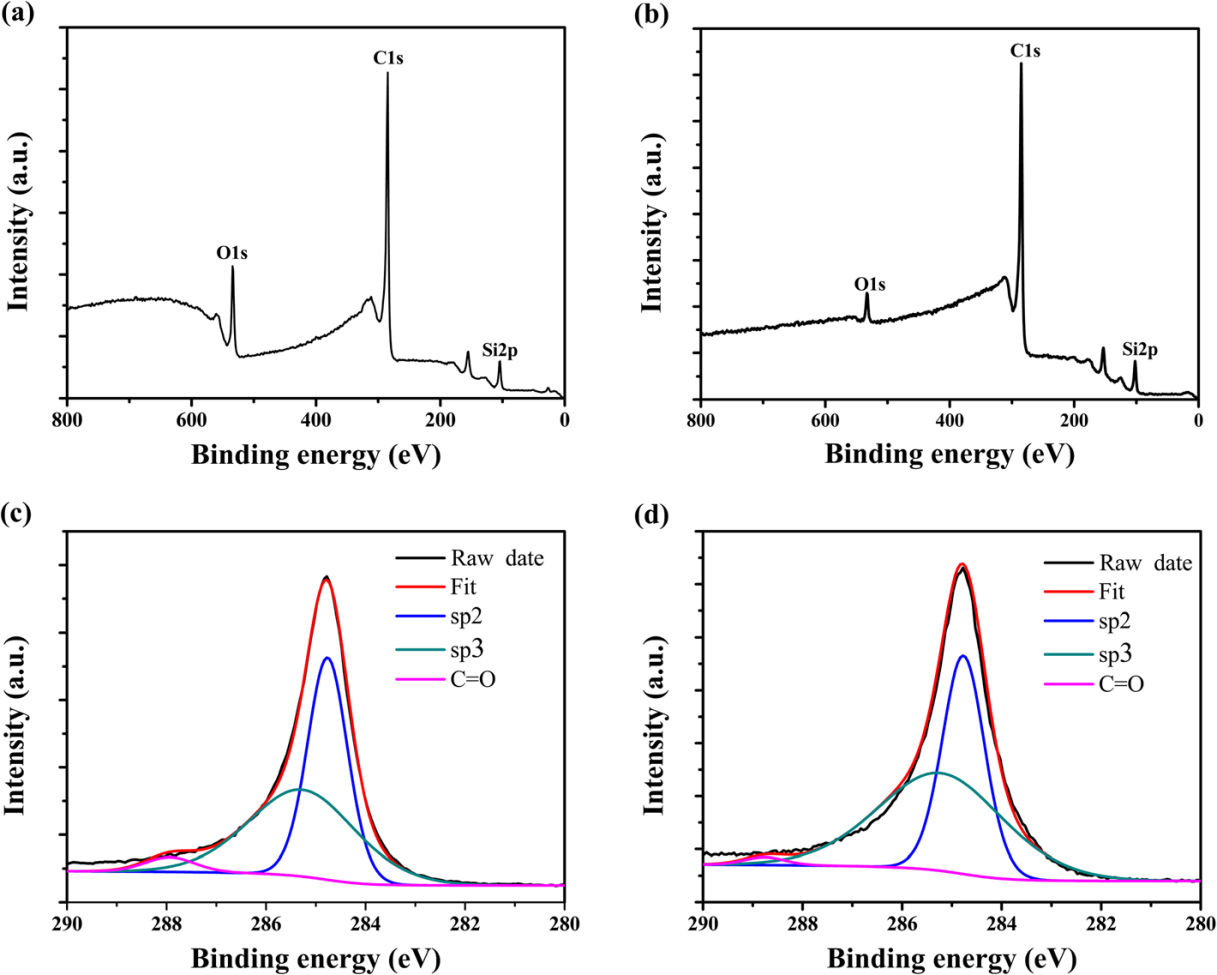
XPS spectra of graphene dendrites grown on SiO_2_ (**a**) and Si (**b**) surface of the SiO_2_/Si substrates. Curve fit of C1s peak of graphene dendrites grown on SiO_2_ (**c**) and Si (**d**) surface of the SiO_2_/Si substrates

X-ray photoelectron spectroscopy (XPS) was also performed to further illustrate the detail structural characterization of the graphene dendrites prepared on the Si and SiO_2_ surface of the SiO_2_/Si substrates (Fig. 5). The peak C1s at about ~ 284 eV is clearly observed on both Si and SiO_2_ surface, which can be assigned to the sp^2^ C-C network [33]. The peaks of O1s at ~ 533 eV and Si2p at ~ 104 eV are also observed, which can be assigned to the SiO_2_/Si substrate [34]. Figure 5c, d show the cure fit of C1s from Fig. 5a, b, respectively. For both cases, the peak C1s can be divided into three peaks. The main peak at ~ 284.7 eV reveals the appearance of sp^2^ hybridization peak of graphene. The peak at ~ 285.3 eV is assigned to the sp^3^-hybridization C-C bonding ascribing to the contamination of natural carbon [35]. The weak peak at ~ 288 eV relates to carbon C=O groups, which probably originate from the oxygen contamination during graphene dendrite growth [36].

**Fig. 6:**
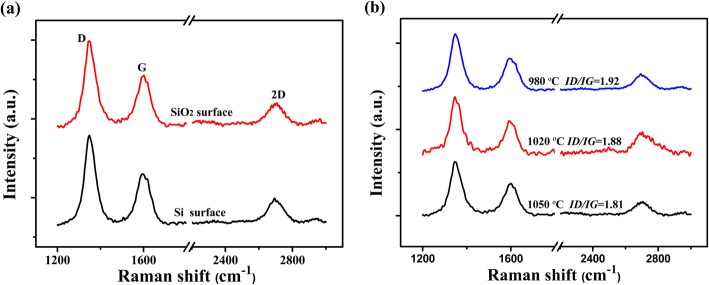
**a** Raman spectra of graphene dendrites grown on both Si and SiO_2_ surface of SiO_2_/Si substrates. **b** Raman spectra of grpahene dendrites grown on Si surface from 980 °C to 1050 °C

Raman spectra were performed to investigate the crystal quality and the number of layers of the graphene dendrites [37–39]. As shown in Fig. 6a, the D peak (~ 1350 cm^−1^), G peak (~ 1580 cm^−1^), and 2D peak (~ 2680 cm^−1^) of graphene are observed on both Si and SiO_2_ surface of the SiO_2_/Si substrates. The G peak is the characteristic of the carbon sp^2^ structure and the 2D peak is the second order of zone boundary phonons. The D peak is a defect-related peak reflecting the disorder of graphene [40–43]. For the graphene dendrites grown on both Si and SiO_2_ surface, the intensity of the G peak is much higher than that of the 2D peak, indicating that graphene dendrites grown on both Si and SiO_2_ surfaces have a multilayered structure. In addition, as the growth temperature increases from 980 to 1050 °C, the peak intensity ratio of *I*_*D*_*/I*_*G*_ decreases from 1.92 to1.81, indicating that the quality of the graphene dendrites was improved with the increase of growth temperature (Fig. 6b) [40–43].

**Fig. 7:**
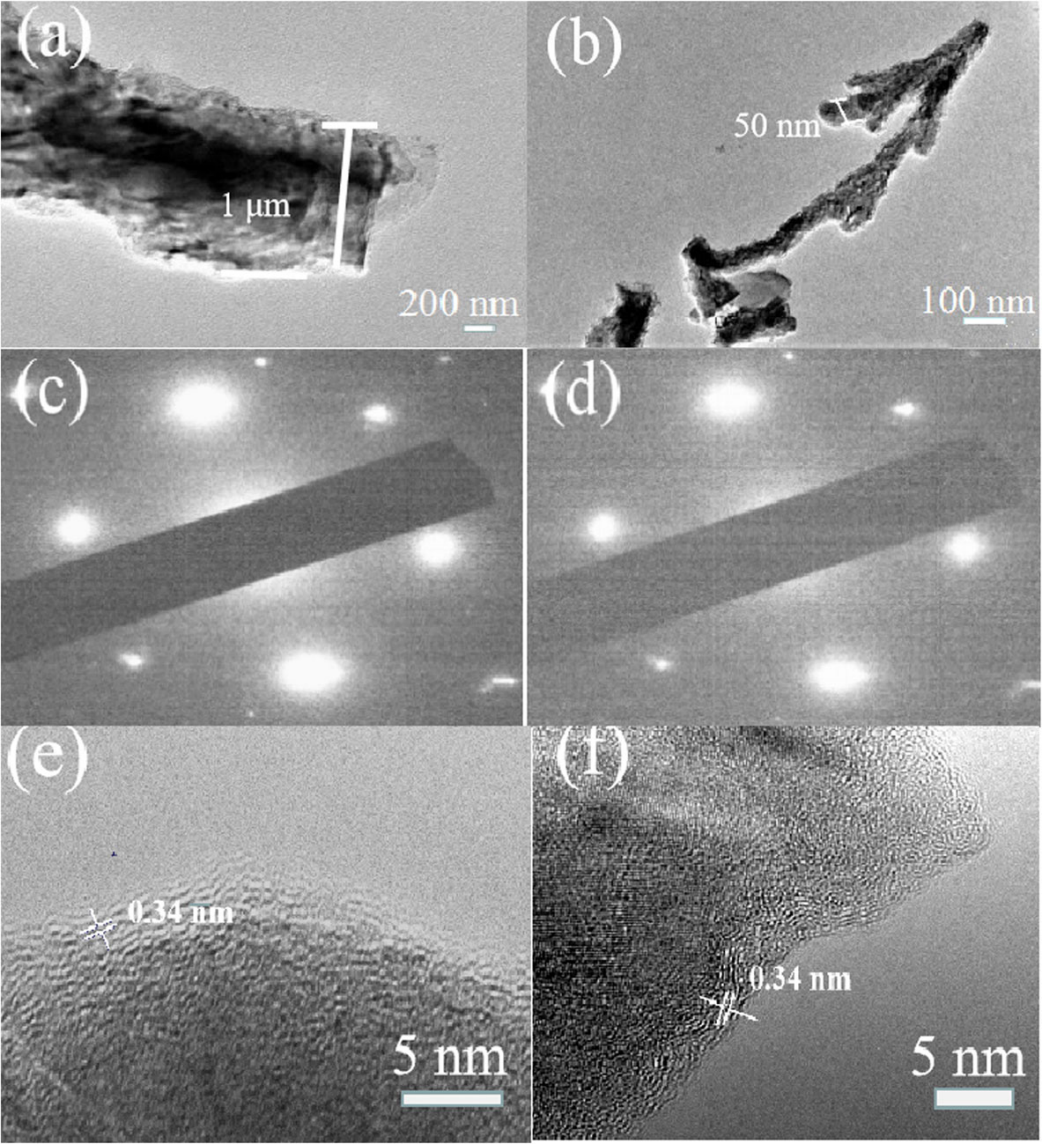
Low-magnification TEM images of the primary (**a**) and the secondary (**b**) graphene dendrites, and the SAED patterns taken from primary (**c**) and the secondary (**d**) graphene dendrites, respectively. High-resolution TEM images of primary (**e**) and the secondary (**f**) graphene dendrites

We further performed TEM and SAED to investigate the detailed structure of graphene dendrites . The samples were transferred to the TEM grid before the TEM imaging. From Fig. 7a, b, we can see that the diameter of the primary and secondary graphene dendrites is about 1 μm and 50 nm, respectively. Figure 7c, d show the SAED patterns from the primary and secondary graphene dendrites, respectively. For both cases, the patterns show a typical 6-fold symmetry of graphene [44, 45]. The high-resolution TEM (HRTEM) images of primary and secondary graphene dendrites taken from the edge of the samples are shown in Fig. 7e, f. For both cases, the HRTEM image shows a multilayer structure, indicating the dendrite is made up of multilayer graphene. This result is consistent with Raman characterization.

**Fig. 8:**
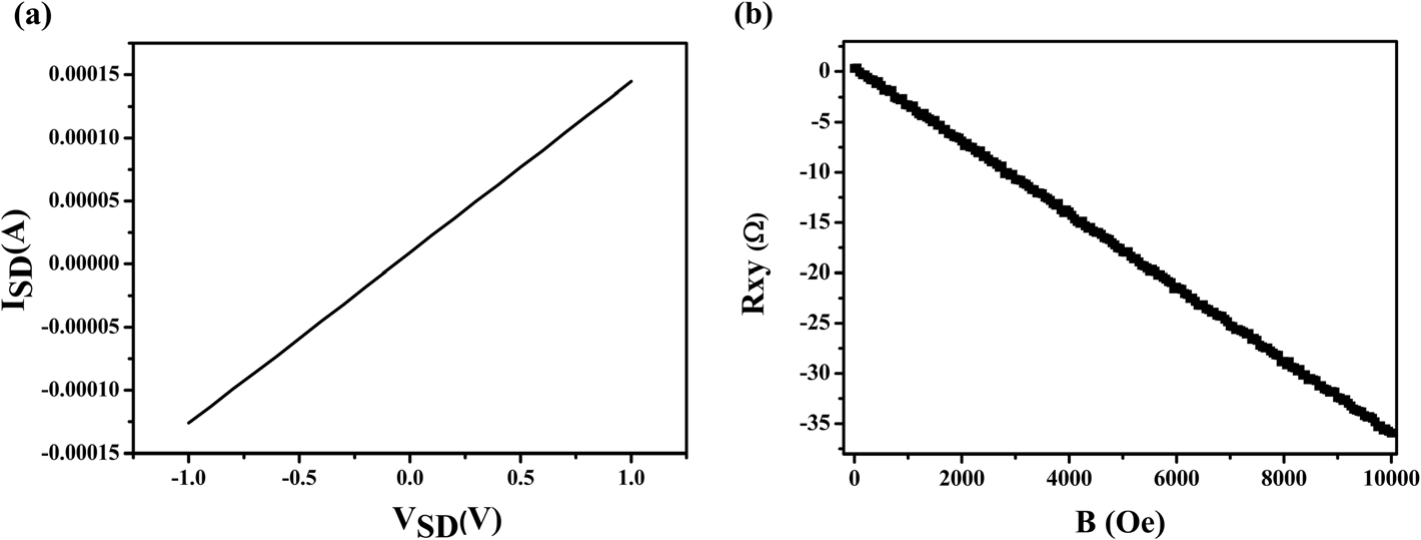
**a** A representative current-voltage (*I*_SD_-*V*_SD_) curves of the graphene dendrites at zero gate voltage. **b** Hall resistances as a function of magnetic field strength for graphene dendrites

The electrical properties of the graphene dendrites were evaluated with a back-gated field-effect transistor (FET). For the electrical measurement, the samples were placed into a probe station. Two tungsten microprobes (10 μm in diameter) were used as the source and the drain electrodes and placed directly on the SiO_2_ surface at two ends of several selected samples of graphene dendrites. Figure 8a shows the linear and reproducible *I*_SD_-*V*_SD_ curves at zero gate voltage, demonstrating the ohmic contact obtained between graphene dendrites and tungsten probes. The resistance *R* of the graphene dendrites is approximately 6110 Ω. The resistivity *ρ* is obtained via the equation:

1 $$ \rho = RS/L $$

Where the *S* and *L* are the cross-section and length of the graphene dendrites. The conductivity *σ* is calculated by the formula:

2 $$ \sigma =1/\rho $$

Based on the above analysis, the electrical conductivity of the dendrites is ~ 286 Scm^−1^.

Electronic transport measurements on the SiO_2_ surface with van der Pauw structure were carried out at room temperature. Hall resistance (*R*_*xy*_) as a function of the magnetic field strength has been shown in Fig. 8b. Hall coefficient *R*_H_ is calculated by the formula:

3 $$ {R}_H=R\mathrm{xy}/B\cdot t $$

Where *t* is the thickness of the sample, and *Rxy* is the longitudinal resistance. Hall coefficient is − 1.2 cm^3^/C.

The resistivity of the graphene dendrites is extracted by the equations:

4 $$ \rho =\frac{\pi \kern0.28em t}{1n2}\cdot \frac{R_{xx-1}+{R}_{xx-2}}{2}\cdot f\left(\frac{R_{xx-1}}{R_{xx-2}}\right) $$

Where *ρ* is the resistivity of the sample, *R*_*xx*_ is the longitudinal resistance, *f* is the van der Pauw factor, and its value is close to 1 thus neglected. The obtained conductivity is ~ 474 S/cm, which is comparable to the value of ~ 286 Scm^−1^ measured by FET.

Furthermore, we further calculate the Hall mobility with the following formula:

5 $$ \mu =\frac{\mid {R}_H\mid }{\rho } $$

The Hall mobility of graphene dendrites is calculated to be ~ 574 cm^2^/Vs, which is much higher than that of the nanocrystalline graphene thin film reported previously [46].

**Fig. 9:**
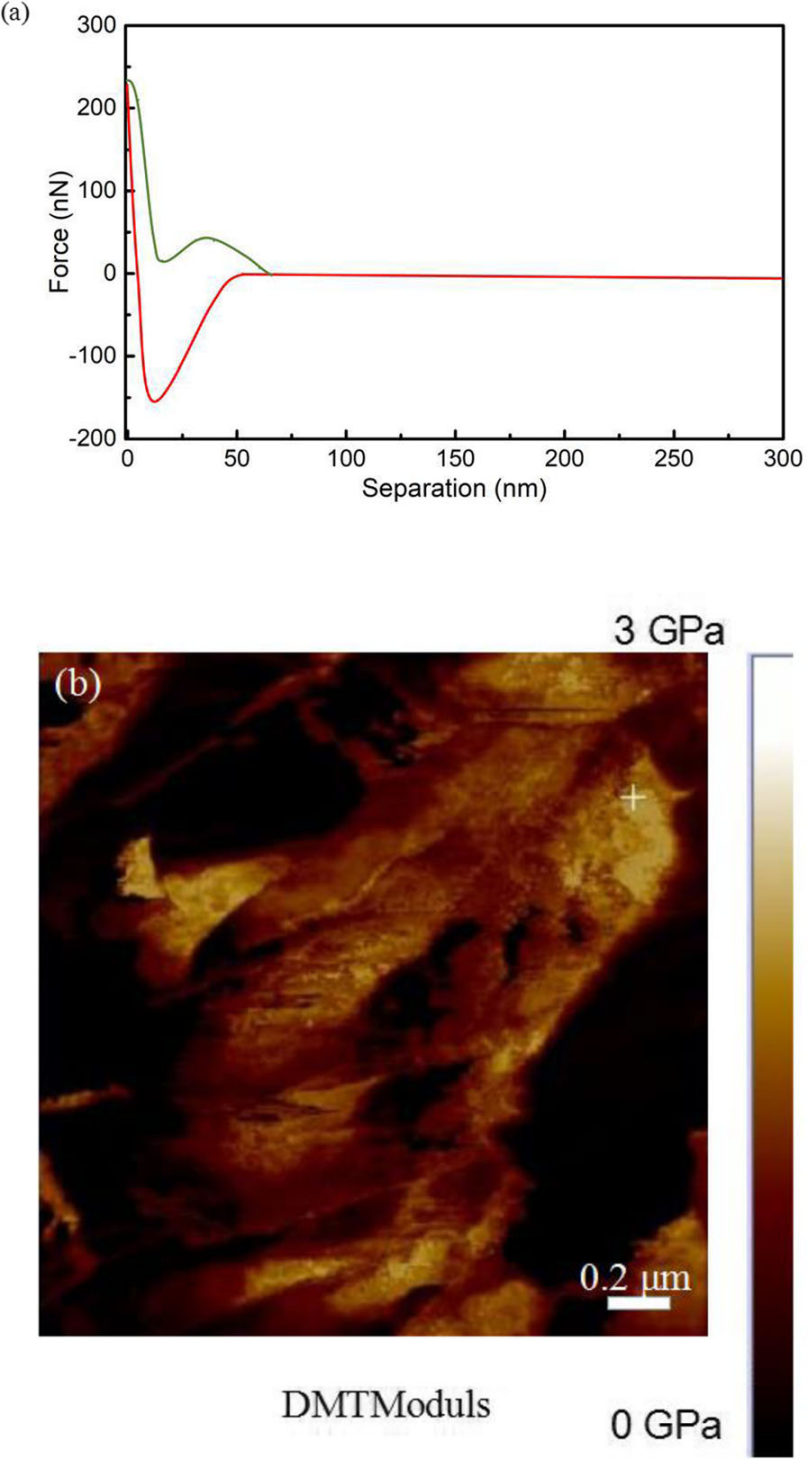
**a** AFM force-displacement curve of graphene dendrites. **b** AFM DMT Modulus image of Young’s modulus of graphene dendrites

To evaluate the mechanical properties of the graphene dendrites, AFM imaging in PFQNM mode was used to investigate Young’s modulus of the graphene dendrites. The measurements were carried out under ambient conditions at room temperature. Figure 9a shows the data that the force plot as a function of the separation, which represents the interaction of one approaching (green line) and retracting (red line) cycle in PFQNM.

In order to obtain Young’s modulus, a fit of the retraction curve was implemented using the Derjaguin-Muller-Toporov (DMT) model [47].

6 $$ F-{F}_{adh}=\frac{4}{3}{E}^{\ast}\sqrt{R{d}^3} $$

where the *F-F*_adh_ represents the force on the cantilever relative to the adhesion force, *R* is the tip end radius, and *d* is the deformation of the sample. The result of the fit is the reduced modulus E*. Young’s modulus can be calculated by the following equation

7 $$ {E}^{\ast }={\left[\frac{1-{V}_S^2}{E_S}+\frac{1-{V}_{tip}^2}{E_{tip}}\right]}^{-1} $$

where the *v*_*s*_ and *v*_tip_ are Poisson’s ratio of the samples and tip, respectively, the *Es* and *E*_tip_ are Young’s modulus the samples and tip, respectively. Sample with a scanning size of 2.0 μm × 2.0 μm was tested. As shown in Fig. 9b, the graphene dendrites are shown in the yellow area of the map. Young’s modulus of graphene dendrites is up to 2.26 GPa obtained from the yellow cross marked region.

We compare the mechanical and electrical properties of different types of graphene-derived materials as shown in Table 1 [11–14, 23]. The conductivity of our graphene dendrite is several orders higher than that of graphene hydrogel and the graphene dendrite produced by the electrochemical method [23]. The value also is comparable to that of the other graphene-derived materials, such as graphene fibers of ~ 10 Scm^−1^ [12] and 2.5 × 10^4^ Sm^−1^ (250 Scm^-1^) [13] and graphene paper of 351 Scm^−1^ [14]. For the mechanical strength, Young’s modulus of graphene dendrites in this work is much higher than graphene hydrogel of~ 450 kPa (~ 4.5 × 10^−4^ GPa), and is also comparable to that of graphene fibers of 420 MPa (0.42 GPa) [12] and ~ 7700 MPa (~ 7.7 GPa) [13]. In comparison with the other graphene-derived materials, the graphene dendrite is more suitable for use in the nanoelectronic device due to nanometer level size in diameter and good compatibility with the existing semiconductor technology.

**Table 1:** The mechanical and electrical properties of different types of graphene-derived materials. This table records Young’s modulus and conductivity of different types of graphene-derived materials (graphene hydrogel, graphene fibers, graphene paper, and graphene dendrites)

Type	Youngʼs modulus	Conductivity	Reference
Graphene hydrogel	~ 450 kPa (~ 4.5×10^−4^ GPa)	5 × 10^−3^ Scm^−1^	[11]
Graphene fibers	420 MPa (0.42 GPa)	~ 10 Scm^−1^	[12]
Graphene fibers	~ 7700 MPa (~ 7.7 GPa)	~ 2.5 × 10^4^ Sm^−1^ (250 Scm^−1^)	[13]
Graphene paper	–	351 Scm^−1^	[14]
Graphene dendrite	–	44 S/m (0.44 Scm^−1^)	[23]
Graphene dendrite	2.26 GPa	~ 286 Scm^−1^	This work

## Conclusions

In this work, we have successfully achieved direct growth of graphene dendrites on both Si and SiO_2_ surfaces on SiO_2_/Si substrates using a CVD method. The morphology of the graphene dendrites can be regulated by the growth temperature and growth time. Raman spectra and TEM analysis indicated that the graphene dendrites have a multilayer structure. The graphene dendrites show excellent electrical properties with the conductivity of ~ 286 Scm^−1^ and Hall mobility of ~ 574 cm^2^(Vs) ^−1^. The graphene dendrites also show a good mechanical performance with Young’s modulus up to 2.26 GPa. The method avoids the need for a complicated and skilled post-growth transfer process and is compatible with current existing semiconductor technology, and thus is very promising in nanoelectronic applications.

## Data Availability

All data generated or analyzed during this study are included in this published article.

## Abbreviations

AFM: Atomic force microscopy
CVD: Chemical vapor deposition
DMT: Derjaguin-Muller-Toporov
EDS: Energy dispersive spectroscopy
PFQNM: PeakForce quantitative nanomechanical mapping
SAED: Selected area electron diffraction pattern
SEM: Scanning electron microscopy
TEM: Transmission electron microscopy
XPS: X-ray photoelectron spectroscopy
